# Mapping Speech-Language Pathology and Audiology Rehabilitation Services Across Saudi Arabia: A Retrospective Cross-Sectional Study

**DOI:** 10.3390/audiolres16030069

**Published:** 2026-05-10

**Authors:** Mohammed F. Alharbi, Ahmad A. Alanazi

**Affiliations:** 1 Department of Speech-Language Pathology and Audiology, College of Medical Rehabilitation Sciences, Taibah University, Al-Madinah Al-Munawarrah 42353, Saudi Arabia; mfmharbi@taibahu.edu.sa; 2 Department of Audiology and Speech Pathology, College of Applied Medical Sciences, King Saud bin Abdulaziz University for Health Sciences, P.O. Box 3660, Riyadh 11481, Saudi Arabia; 3 King Abdullah International Medical Research Center, Riyadh 11481, Saudi Arabia; 4 Ministry of National Guard Health Affairs, Riyadh 11481, Saudi Arabia

**Keywords:** audiology, health data, rehabilitation services, speech-language pathology, Saudi Arabia, service utilization

## Abstract

**Background:** Speech-language pathology (SLP) and audiology services are essential components of multidisciplinary rehabilitation, particularly for individuals with developmental, neurological, and communication-related disorders. National-level data describing the distribution and utilization of these services in Saudi Arabia remain limited. This study aimed to examine national patterns of rehabilitation service utilization, with a focus on SLP and audiology services in comparison to other rehabilitation specialties. **Methods:** A retrospective cross-sectional analysis was conducted using publicly available national open data released by the Saudi Ministry of Health (MOH). Aggregated rehabilitation service encounters (n = 1,872,328 to 1,930,695) from 2023–2024 were analyzed by specialty, geographic region, sector (MOH clusters versus private sector), and pediatric age groups. Descriptive statistics were used to characterize utilization patterns and regional variation. **Results:** Rehabilitation services were widely delivered across both public and private sectors, with physiotherapy representing the largest share of encounters. SLP and audiology services contributed a smaller proportion of total rehabilitation encounters compared to other specialties. Service distribution varied regionally, with higher volumes concentrated in major urban areas including Riyadh, Makkah, and the Eastern Region. Pediatric service encounters were highest in early childhood (ages 3–7), with SLP and audiology services forming a consistent component of rehabilitation during this period. **Conclusions:** This study provides a descriptive overview of rehabilitation service utilization in Saudi Arabia, highlighting the distribution of SLP and audiology services relative to other specialties and across regions. Findings emphasize the importance of addressing regional variation, supporting workforce development, and enhancing national rehabilitation data systems to inform planning and ensure comprehensive access to communication and hearing services.

## 1. Introduction

Communication, swallowing, hearing, and balance functions are essential for health, educational attainment, psychological and social participation, and quality of life across the lifespan [1]. Disorders affecting communication, swallowing, hearing, and balance are highly prevalent worldwide and are frequently associated with neurological conditions, developmental disorders, head and neck diseases, and aging-related processes [2]. For example, hearing loss alone is recognized as one of the leading causes of disability globally, with an estimated 430 million people currently requiring rehabilitation, a number projected to rise significantly by 2050 [3]. Likewise, speech and language disorders are common contributors to childhood disability and long-term functional impairment when not identified and treated early [4].

Medical rehabilitation services play a central role in reducing disability and improving functional outcomes and subsequently, the quality of life of individuals with acute, chronic, and developmental conditions [5]. These services typically encompass multiple disciplines, including speech-language pathology (SLP), audiology, physical therapy (PT), occupational therapy (OT), respiratory therapy (RT), and prosthetics and orthotics (PO). International best practices emphasize multidisciplinary, patient- and family-centered rehabilitation models in which speech-language pathologists and audiologists are integral members of care teams [5]. However, access to rehabilitation services and the distribution of rehabilitation specialties vary considerably across healthcare systems and countries, often reflecting workforce availability and referral patterns rather than actual population need.

In Saudi Arabia, demand for rehabilitation services has grown in recent years due to population increases, improved survival following acute medical conditions, and greater recognition of developmental disabilities within clinical and educational systems [5]. National health transformation efforts under Saudi Vision 2030 prioritize improvements in the quality and effectiveness of healthcare services, including expansion of rehabilitation services and equitable access across regions [6]. Despite these policy priorities, recent workforce analyses indicate that the provision of rehabilitation services in Saudi Arabia remains imbalanced, with notable variation in workforce distribution across the regions, and is not related to the need [7]. For example, physiotherapist and occupational therapist ratios per 10,000 population differ widely across administrative regions, and workforce distribution does not consistently align with the estimated need [7]. These findings highlight the complexity of aligning rehabilitation services with population demand in the country.

SLP and audiology services are particularly relevant to several high-burden clinical populations within the Saudi healthcare system. Neurological conditions such as stroke, traumatic brain injury, neurodegenerative diseases, and congenital hearing loss often result in communication, swallowing, and/or auditory and balance impairments that necessitate integrated SLP and audiology services. For example, according to the Saudi General Authority for Statistics, 1.4% and 1.1% of the total Saudi population experience mild and severe to extreme hearing and communication difficulties, respectively [8]. Despite this relevance, emerging evidence suggests that SLP and audiology services may be underrepresented within national rehabilitation service delivery, with adequate availability largely confined to major cities, such as Riyadh and Jeddah [1,9]. Awareness and understanding of the roles of speech-language pathologists and audiologists among healthcare professionals in Saudi Arabia have improved, but referral patterns and integration within multidisciplinary teams remain inconsistent [10]. Additionally, studies focusing on service quality and availability, for example, in educational and clinical settings, underscore the need to strengthen and expand SLP and audiology services, particularly for children with learning disabilities and communication disorders [1,11]. Academic and professional capacity is expanding, with new undergraduate and graduate programs in SLP and audiology being developed to address workforce shortages [5].

That said, the absence of comprehensive, linked national rehabilitation data has hindered systematic assessment of whether current service provision aligns with actual clinical demand. National open administrative health data presents a valuable opportunity to examine real-world patterns of rehabilitation service utilization and identify potential gaps in care delivery. Comparative analysis across rehabilitation specialties and geographic regions can provide insight into service distribution, intensity of use, and alignment between clinical demand and available resources. Such analyses are particularly important for communication and audiology services, which are often less visible in health system planning despite their broad functional impact. Therefore, this study aimed to examine national patterns of rehabilitation service utilization in Saudi Arabia, with a specific focus on SLP and audiology services compared to other rehabilitation specialties (e.g., PT, OT, PO, and RT). By leveraging national open data, this study seeks to inform workforce planning, service integration, and policy development aimed at strengthening and equitably expanding communication and hearing rehabilitation services across Saudi Arabia.

## 2. Methods

### 2.1. Study Design

This study employed a retrospective descriptive and comparative analysis of national open administrative health data to examine patterns of medical rehabilitation service utilization in Saudi Arabia. The analysis focused on comparing SLP and audiology services with other rehabilitation specialties across regions and clinical service indicators.

### 2.2. Data Sources

Data were obtained from publicly available national open datasets named “Statistical Yearbook 2023–2024”, recently released by the Ministry of Health (MOH) in Saudi Arabia [12]. These datasets included aggregated information on rehabilitation service encounters, clinical service categories (e.g., neurology and developmental services), pediatric rehabilitation utilization by age group, and regional distribution of rehabilitation services among MOH clusters and the private sector. The primary dataset used in this analysis was compiled from the reported information within the national open data platform. All data were fully anonymized, aggregated at the regional and service level, and did not include any personal identifiers.

### 2.3. Study Variables

Rehabilitation services were categorized according to the available data, with the focus on SLP, audiology, and other rehabilitation services (e.g., PT and OT). Key utilization indicators included the total number of rehabilitation encounters by specialty, distribution of encounters across regions, the comparison of rehabilitation services between the MOH facilities and the private sector, and rehabilitation utilization by pediatric age groups.

### 2.4. Data Management

Data were imported into Microsoft Excel for inspection, cleaning, and preparation. To improve transparency and reproducibility, data processing followed a structured pipeline consisting of three main stages: identification, harmonization, and aggregation. In the identification stage, relevant datasets were selected from the national open data platform based on their alignment with the study objectives. Selection criteria included relevance to healthcare service indicators, completeness of variables, and the availability of regional and specialty-level information. Given the structure of the datasets, which included stacked formats, relevant data segments were isolated to retain only analyzable content.

During the harmonization stage, variables were standardized across datasets to ensure consistency. This involved aligning variable names across Arabic and English fields, reconciling differences in terminology, and ensuring consistent categorization of specialties and regions. Data cleaning procedures were also applied, including the removal of summary rows, non-numerical entries, duplicated headers, and incomplete or ambiguous labels. In the final aggregation stage, the cleaned and harmonized data were combined to construct the analytic dataset. Service counts were summarized at the specialty and regional levels, ensuring consistency in definitions and units across datasets. Due to the aggregated nature of the data, direct linkage between individual diagnoses, healthcare providers, and patient encounters was not possible.

### 2.5. Data Analysis

Analyses included calculation of total and relative rehabilitation service utilization by specialty, with comparative assessment of SLP and audiology utilization relative to other rehabilitation specialties, across regions, and within pediatric age groups. Results were reported using descriptive, non-parametric statistics, including frequencies and percentages, as the purpose of the study was to explore national patterns and disparities rather than test causal hypotheses. Percentages were calculated using clearly defined denominators, specifically the total number of services within each region or specialty category, depending on the analysis. Comparisons across regions and specialties were conducted using proportional distributions rather than inferential statistical tests.

Data analysis was performed using IBM SPSS Statistics for Windows, Version 29.0. Prior to analysis, data quality procedures were implemented to ensure consistency and reproducibility. Missing and inconsistent data were addressed through the exclusion of incomplete entries, removal of non-numerical values, and standardization of variable labels during the data cleaning process. Analyses were conducted on available-case data following these procedures, providing a reliable summary of the aggregated dataset.

### 2.6. Ethical Considerations

This study used publicly available, de-identified data so that it is impossible to link a record to a particular individual and did not involve human subjects or access to confidential patient information. This study was approved by the Institutional Review Board of Taibah University (#TU-RHB-26-02-007). The procedures adhered to the applicable ethical guidelines of the Declaration of Helsinki.

## 3. Results

### 3.1. Rehabilitation Services by Specialty and Region

**Figure 1:**
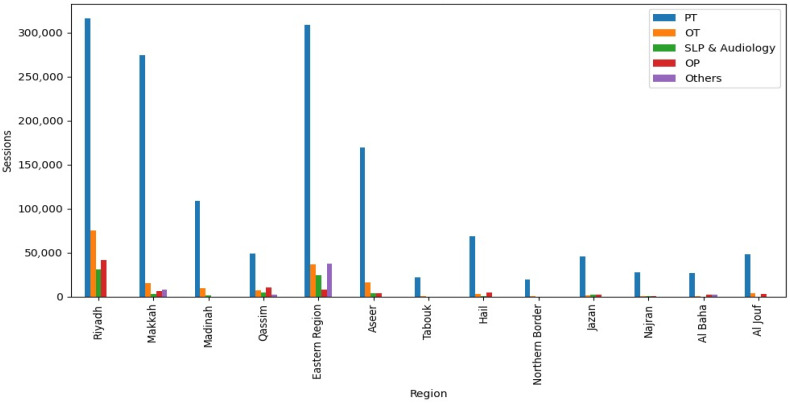
The distribution of rehabilitation services by type and region among the Ministry of Health clusters in Saudi Arabia.

**Figure 2:**
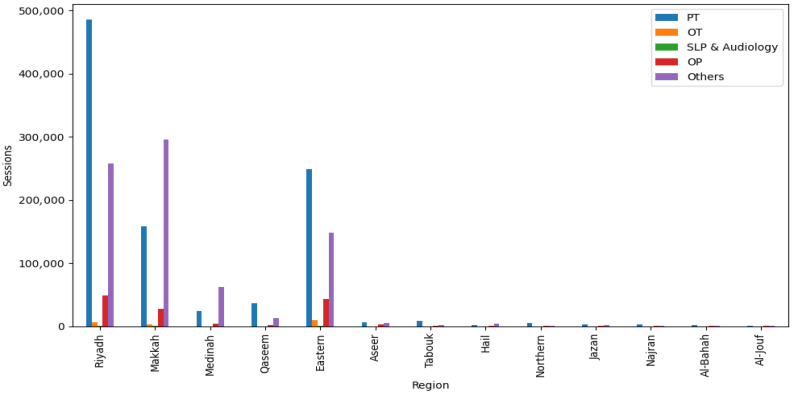
The distribution of rehabilitation services by type and region in the private sector in Saudi Arabia.

A total of 1,872,328 rehabilitation sessions were provided by MOH clusters, compared to 1,930,695 sessions delivered by the private sector. Although the overall service volumes were comparable, marked differences were observed in the distribution of services by specialty and region. Figure 1 and Figure 2 show the distribution of rehabilitation service encounters by the type of specialty and region among MOH clusters and the private sector in Saudi Arabia. PT represented the largest proportion of rehabilitation services in both sectors. MOH clusters delivered 1,486,286 PT sessions (79.4%), whereas the private sector provided 985,321 sessions (51%). OT services followed a similar pattern, with MOH clusters providing 174,328 sessions (9.3%) compared with 19,810 sessions (1%) in the private sector. SLP and audiology utilization was substantially lower, with MOH clusters reporting 74,469 sessions (4%) and the private sector only 1450 sessions. In contrast, the private sector exceeded MOH clusters in outpatient PT, delivering 132,578 sessions (6.8%) versus 85,524 sessions (4.5%) in MOH facilities. Other rehabilitation services, such as RT, were predominantly provided by the private sector, with 791,536 sessions (41%) compared with 51,721 sessions (2.7%) in MOH clusters.

To account for differences in population size across regions, service utilization rates per 100,000 population were calculated for each region. For example, Riyadh Province recorded 464,224 MOH sessions, corresponding to approximately 5402 sessions per 100,000 population, while Makkah Province had 375,000 sessions (approximately 4830 per 100,000 population) and the Eastern Province had 277,500 sessions (approximately 5420 per 100,000 population). Smaller or less densely populated regions, such as Tabuk, Northern Borders, Najran, and Al Baha, had lower per-capita rates, ranging from approximately 1500 to 2800 sessions per 100,000 population, despite MOH clusters providing the majority of services in these areas. These results highlight relative patterns of service distribution by specialty and region. They are presented as descriptive trends rather than direct indicators of unmet need, with population-adjusted rates providing context for interpreting regional differences in service provision.

### 3.2. Rehabilitation Services Among Pediatric Age Groups

Analysis of children’s age-group data demonstrated a clear pediatric utilization peak, particularly during early childhood. Table 1 shows rehabilitation services across children’s age groups. The average number of sessions per child reported in Table 1 was directly obtained from the dataset and was not calculated by the authors. The dataset includes aggregated service counts and estimated numbers of pediatric recipients, but does not contain individual-level identifiers. Therefore, the reported average may reflect the total number of sessions divided by the estimated number of children. The use of rehabilitation services increased progressively from age one year. The distribution of rehabilitation services by age group demonstrates clear age-related patterns in service utilization across all specialties. Overall, PT represented the highest volume of sessions in every age category, followed by OT, then SLP and audiology, while the others category consistently accounted for a minimal proportion of total sessions. Service utilization increased progressively with age, beginning with relatively low session counts in children aged 1 year and rising steadily through middle childhood. PT sessions increased from 550 sessions at age 1 to more than 4000 sessions by age 6, indicating a marked escalation in physical rehabilitation needs during early childhood. A similar upward trend was observed for OT, SLP, and audiology services, particularly between ages 2 and 7 years.

Children aged 3 and 4 years demonstrated the highest average number of sessions per child (3.3 sessions), suggesting greater service intensity during this developmental period. This peak likely reflects increased identification of developmental delays and early intervention needs during preschool years. Following this peak, the average number of sessions per child gradually declined, stabilizing around 2.0–2.2 sessions per child from ages 7 to 11. The highest absolute service utilization was observed in the 12-year-old and older age group, which accounted for the largest share of PT (6381 sessions), OT (2450 sessions), and SLP and audiology sessions (2972 sessions). Despite these high absolute numbers, the average number of sessions per child in this age group was relatively lower (1.9), indicating broader service coverage with lower intensity per child compared with younger age groups. Across all age groups combined, a total of 39,480 PT sessions, 18,638 OT sessions, and 15,457 SLP and audiology sessions were recorded, with an overall average of 2.3 sessions per child. These findings highlight early childhood as a period of higher rehabilitation intensity, followed by sustained but less intensive service utilization into adolescence. Despite this high service intensity, SLP and audiology services did not demonstrate proportional representation in visit counts, suggesting that many children receiving long-term rehabilitation may not be receiving comprehensive communication and hearing-related interventions.

## 4. Discussion

This national analysis of open rehabilitation data provides a descriptive overview of rehabilitation service utilization in Saudi Arabia. While rehabilitation services are widely used, the distribution of services varies across specialties, regions, and pediatric age groups, with SLP and audiology services reported less frequently than PT and OT. These patterns reflect relative differences in service delivery rather than direct measures of unmet clinical need.

### 4.1. Rehabilitation Services by Specialty and Region

This study demonstrates clear differences in rehabilitation service distribution across specialties and regions. PT accounted for the largest proportion of rehabilitation sessions in both MOH clusters and the private sector, consistent with previous national and international reports identifying PT as a cornerstone of rehabilitation service delivery. PT services in Saudi Arabia are typically the most established and widely available, supported by a larger workforce and broader referral pathways compared with other rehabilitation specialties [5,13]. The higher volume of PT, OT, and SLP/audiology services in MOH clusters compared with the private sector reflects the central role of the public healthcare system in providing comprehensive rehabilitation care. Alanazi et al. reported that public rehabilitation services remain the primary providers of multidisciplinary care, particularly for complex and long-term conditions, due to broader coverage and integration into government health systems [5].

Regional analyses showed that Riyadh, Makkah, and the Eastern Region recorded the highest rehabilitation service volumes across most specialties, reflecting population density, healthcare infrastructure concentration, and availability of specialized rehabilitation facilities [13,14,15]. Peripheral regions such as Tabuk, Northern Border, Najran, and Al Jouf had lower service volumes and greater reliance on MOH clusters, reflecting limited private sector participation. These descriptive patterns are consistent with prior studies documenting geographic disparities in rehabilitation access, often related to workforce shortages and reduced facility capacity outside metropolitan areas [13,16,17].

### 4.2. Rehabilitation Services Among Pediatric Age Groups

Analysis of pediatric utilization revealed increasing rehabilitation service use from infancy through adolescence, with PT consistently representing the most utilized service across all age groups. These findings align with pediatric rehabilitation literature emphasizing the high prevalence of motor impairments and functional limitations requiring PT intervention [18]. PT is often the first rehabilitation service accessed by children with developmental, neurological, or musculoskeletal conditions, explaining its dominance across age groups [19].

The highest average number of sessions per child was observed among children aged 3–4 years, suggesting increased rehabilitation intensity during early childhood. This trend is consistent with early intervention priorities, as this developmental period is critical for identifying delays and initiating services associated with improved functional outcomes [18,20]. Although children aged 12 years and older accounted for the highest absolute number of sessions, lower average sessions per child likely reflect broader service coverage with reduced intensity, consistent with long-term management or maintenance-focused rehabilitation. Early intervention programs in Saudi Arabia have expanded in recent years, particularly within MOH facilities, contributing to increased service utilization in preschool-aged children [19]. Similar age-related trends have been reported internationally, where rehabilitation intensity peaks in early childhood and gradually stabilizes during adolescence [20,21].

Across pediatric age groups, SLP and audiology services were reported less frequently than PT and OT. These descriptive patterns reflect relative service distribution in the dataset, without implying quantifiable unmet need. Prior studies have documented limited availability of speech-language pathologists and audiologists in primary healthcare and school settings [1,5], which may contribute to these patterns, but our findings cannot confirm gaps relative to population-level clinical need.

### 4.3. Implications for Health System Planning

These descriptive findings inform health system planning by identifying patterns of service distribution that may guide future resource allocation. Expanding SLP and audiology capacity, particularly in regions with lower reported service volumes, alongside integration into multidisciplinary services and the development of national SLP and audiology databases, can support more balanced service delivery. Workforce development, improved referral systems, and integration of rehabilitation services across care settings are priorities identified in national and global rehabilitation frameworks [16,22]. Regional differences in service volumes also highlight the potential role of innovative delivery models, such as tele-rehabilitation and community-based programs, to improve access in remote regions. Recent Saudi studies have demonstrated the feasibility and acceptability of tele-rehabilitation for pediatric and outpatient populations [15,23]. These initiatives align with Saudi Vision 2030 goals related to healthcare accessibility, quality, and workforce development [6].

### 4.4. Study Limitations

This study has several limitations. First, the analysis relied on open data that were not originally collected for research purposes, limiting the availability of detailed clinical information such as diagnoses, severity of impairment, and individual patient outcomes. Second, rehabilitation encounters could not be directly linked to specific providers or professional disciplines, which may have resulted in misclassification across service categories. Third, the use of aggregated regional data precluded assessment of individual-level utilization patterns or longitudinal service trajectories. Finally, incomplete national workforce data limits the ability to directly evaluate provider-to-population ratios for SLP and audiology services. Despite these limitations, the study provides valuable national-level insights into rehabilitation service utilization and identifies critical gaps in communication and hearing rehabilitation services.

## 5. Conclusions

This national analysis of open rehabilitation data provides a comprehensive, descriptive overview of the utilization and distribution of rehabilitation services across Saudi Arabia. PT and OT services were widely delivered across regions, reflecting established service availability. In contrast, SLP and audiology services were comparatively less represented, particularly among pediatric populations. Population-adjusted rates highlight regional variability in service provision, underscoring relative differences rather than direct evidence of unmet need. These descriptive patterns provide critical insight into the structure and scope of rehabilitation services nationally, revealing areas where service capacity may be comparatively lower.

The observed distributional patterns have important implications for policy and strategic planning. Workforce development initiatives, particularly targeting underrepresented specialties such as SLP and audiology, are essential to ensure more balanced service availability. Integrating rehabilitation services across public and private sectors, combined with the development of standardized national rehabilitation data systems, can enhance equitable access and facilitate data-driven decision-making. Furthermore, these findings provide a foundation for evidence-informed strategies aimed at improving the equity, comprehensiveness, and efficiency of rehabilitation care across Saudi Arabia and underscore the need for future research incorporating population-adjusted benchmarks, condition-specific prevalence, and service demand modeling to better inform resource allocation, optimize service delivery, and strengthen national rehabilitation planning.

## Figures and Tables

**Table 1:** Age-group distribution of rehabilitation services.

Age Group (Year)	PT	OT	SLP and Audiology	Others	Average Number of Sessions per Child
1	550	344	89	5	1.8
2	3392	1940	1153	147	1.9
3	3220	1792	1250	72	3.3
4	3173	1566	1029	30	3.3
5	3628	1617	921	43	2.6
6	4201	2012	1473	64	2.2
7	3587	1729	1497	32	2.1
8	3331	1571	1329	34	2.0
9	3050	1381	1131	30	2.1
10	2536	1107	1483	57	2.2
11	2431	1129	1130	24	2.1
>12	6381	2450	2972	45	1.9
TOTAL	39,480	18,638	15,457	583	2.3

## Data Availability

The original contributions presented in the study are included in the article; further inquiries can be directed to the corresponding author. Data can be accessed from publicly available national open datasets named “Statistical Yearbook 2023–2024”, recently released by the Ministry of Health (MOH) in Saudi Arabia.
